# Supplementary material for: Developing and evaluating the impact of a small group communication programme in improving the entrepreneurial competence and economic self-efficacy of smallholder farmers with art skills
Source: PLoS One. 2023 Nov 2;18(11):e0292640. doi: 10.1371/journal.pone.0292640 (PMC10621803; doi:10.1371/journal.pone.0292640)
Supplement: Attachment — Submitted filename: PONE-D-23-15217.pdf [file pone.0292640.s001.pdf]

PLOS ONE

Developing and evaluating the impact of a small group communication programme in improving the entrepreneurial competence and economic self-efficacy of smallholder farmers with art skills

--Manuscript Draft--

**Manuscript Number:**	PONE-D-23-15217
**Article Type:**	Research Article
**Full Title:**	Developing and evaluating the impact of a small group communication programme in improving the entrepreneurial competence and economic self-efficacy of smallholder farmers with art skills
**Short Title:**	impact of a small group communication programme
**Corresponding Author:**	Verlumun Celestine Gever<br>University of Nigeria<br>Nsukka, Nigeria, Enugu NIGERIA
**Keywords:**	art skills; economic self-efficacy; smallholder farmer; entrepreneurial competence small group communication
**Abstract:**	Objective<br><br>The aim of this study was to develop and test the effectiveness of a small group communication in improving the entrepreneurial competence and economic self-efficacy of smallholder farmers with art skills.<br><br>Method<br><br>The researchers in study 1 conducted interviews among experts and developed a small group communication programme to be delivered in 24 months. In study 2, a quasi-experiment was conducted involving 540 smallholder farmers in Nigeria to test the impact of the developed programme.<br><br>Results<br><br>The result showed that smallholder farmers with art skills who received the small group communication programme reported a significant improvement in their entrepreneurial competence and economic self-efficacy compared to smallholder farmers who did not receive the programme. A follow-up assessment after two years revealed the steady effectiveness of the developed programme. The result achieved a statistical significance even after controlling for the type of art skills.<br><br>Originality<br><br>This study has developed and tested a small group communation programme that could be useful in assisting farmers with art skills to improve on their entrepreneurial competence and economic self-efficacy of smallholder farmers with art skills.
**Order of Authors:**	Verlumun Celestine Gever
**Additional Information:**	
**Question**	**Response**
**Financial Disclosure**<br><br>Enter a financial disclosure statement that describes the sources of funding for the work included in this submission. Review the submission guidelines for detailed requirements. View published research	The study was not funded.

Powered by Editorial Manager® and ProduXion Manager® from Aries Systems Corporation

articles from *PLOS ONE* for specific examples.<br><br>This statement is required for submission and **will appear in the published article** if the submission is accepted. Please make sure it is accurate.<br><br>**Unfunded studies**<br>Enter: *The author(s) received no specific funding for this work.*<br><br>**Funded studies**<br>Enter a statement with the following details:<br>• Initials of the authors who received each award<br>• Grant numbers awarded to each author<br>• The full name of each funder<br>• URL of each funder website<br>• Did the sponsors or funders play any role in the study design, data collection and analysis, decision to publish, or preparation of the manuscript?<br>• **NO** - Include this sentence at the end of your statement: *The funders had no role in study design, data collection and analysis, decision to publish, or preparation of the manuscript.*<br>• **YES** - Specify the role(s) played.<br><br>* typeset	
**Competing Interests**<br><br>Use the instructions below to enter a competing interest statement for this submission. On behalf of all authors, disclose any competing interests that could be perceived to bias this work—acknowledging all financial support and any other relevant financial or non-financial competing interests.<br><br>This statement is required for submission and **will appear in the published article** if the submission is accepted. Please make sure it is accurate and that any funding sources listed in your Funding Information later in the submission form are also declared in your Financial Disclosure statement.	We do not have a competing interest.

Powered by Editorial Manager® and ProduXion Manager® from Aries Systems Corporation

View published research articles from *PLOS ONE* for specific examples.<br><br>**NO authors have competing interests**<br><br>Enter: *The authors have declared that no competing interests exist.*<br><br>**Authors with competing interests**<br><br>Enter competing interest details beginning with this statement:<br><br>*I have read the journal's policy and the authors of this manuscript have the following competing interests: [insert competing interests here]*<br><br>* typeset	
**Ethics Statement**<br><br>Enter an ethics statement for this submission. This statement is required if the study involved:<br><br>• Human participants<br>• Human specimens or tissue<br>• Vertebrate animals or cephalopods<br>• Vertebrate embryos or tissues<br>• Field research<br><br>Write "N/A" if the submission does not require an ethics statement.<br><br>General guidance is provided below. Consult the submission guidelines for detailed instructions. **Make sure that all information entered here is included in the Methods section of the manuscript.**	NA

**Format for specific study types**<br><br>**Human Subject Research (involving human participants and/or tissue)**<br>• Give the name of the institutional review board or ethics committee that approved the study<br>• Include the approval number and/or a statement indicating approval of this research<br>• Indicate the form of consent obtained (written/oral) or the reason that consent was not obtained (e.g. the data were analyzed anonymously)<br><br>**Animal Research (involving vertebrate animals, embryos or tissues)**<br>• Provide the name of the Institutional Animal Care and Use Committee (IACUC) or other relevant ethics board that reviewed the study protocol, and indicate whether they approved this research or granted a formal waiver of ethical approval<br>• Include an approval number if one was obtained<br>• If the study involved *non-human primates*, add *additional details* about animal welfare and steps taken to ameliorate suffering<br>• If anesthesia, euthanasia, or any kind of animal sacrifice is part of the study, include briefly which substances and/or methods were applied<br><br>**Field Research**<br><br>Include the following details if this study involves the collection of plant, animal, or other materials from a natural setting:<br>• Field permit number<br>• Name of the institution or relevant body that granted permission	
**Data Availability**<br><br>Authors are required to make all data underlying the findings described fully available, without restriction, and from the time of publication. PLOS allows rare exceptions to address legal and ethical concerns. See the PLOS Data Policy and FAQ for detailed information.	Yes - all data are fully available without restriction

A Data Availability Statement describing where the data can be found is required at submission. Your answers to this question constitute the Data Availability Statement and **will be published in the article**, if accepted.<br><br>**Important:** Stating 'data available on request from the author' is not sufficient. If your data are only available upon request, select 'No' for the first question and explain your exceptional situation in the text box.<br><br>Do the authors confirm that all data underlying the findings described in their manuscript are fully available without restriction?	
**Describe where the data may be found in full sentences. If you are copying our sample text, replace any instances of XXX with the appropriate details.**<br><br>• If the data are **held or will be held in a public repository**, include URLs, accession numbers or DOIs. If this information will only be available after acceptance, indicate this by ticking the box below. For example: *All XXX files are available from the XXX database (accession number(s) XXX, XXX.).*<br>• If the data are all contained **within the manuscript and/or Supporting Information files**, enter the following: *All relevant data are within the manuscript and its Supporting Information files.*<br>• If neither of these applies but you are able to provide **details of access elsewhere**, with or without limitations, please do so. For example:<br><br>*Data cannot be shared publicly because of [XXX]. Data are available from the XXX Institutional Data Access / Ethics Committee (contact via XXX) for researchers who meet the criteria for access to confidential data.*<br><br>*The data underlying the results presented in the study are available from (include the name of the third party*	Data are available from the corresponding author.

and contact information or URL).<br>• This text is appropriate if the data are owned by a third party and authors do not have permission to share the data.<br><br>* typeset	
Additional data availability information:	

Powered by Editorial Manager® and ProduXion Manager® from Aries Systems Corporation

Click here to access/download;Manuscript;Main doc art skills.docx

Developing and evaluating the impact of a small group communication programme in improving the entrepreneurial competence and economic self-efficacy of smallholder farmers with art skills

Abstract

Objective: The aim of this study was to develop and test the effectiveness of a small group communication in improving the entrepreneurial competence and economic self-efficacy of smallholder farmers with art skills.

Method: The researchers in study 1 conducted interviews among experts and developed a small group communication programme to be delivered in 24 months. In study 2, a quasi-experiment was conducted involving 540 smallholder farmers in Nigeria to test the impact of the developed programme.

Results: The result showed that smallholder farmers with art skills who received the small group communication programme reported a significant improvement in their entrepreneurial competence and economic self-efficacy compared to smallholder farmers who did not receive the programme. A follow-up assessment after two years revealed the steady effectiveness of the developed programme. The result achieved a statistical significance even after controlling for the type of art skills.

Originality: This study has developed and tested a small group communation programme that could be useful in assisting farmers with art skills to improve on their entrepreneurial competence and economic self-efficacy of smallholder farmers with art skills.

Keywords: art skills; economic self-efficacy; smallholder farmer; entrepreneurial competence small group communication

Introduction

Smallholder farmers are those with ownership of small plots of land who also engage in farming on a small scale using labour from family members. Sabo *et al.* (2017) reveal that 80% of Nigerian farmers belong to this category and contribute significantly to addressing the food need of the nation. Despite the contribution of smallholder farmers to the food security of many developing countries, they have remained largely poor (Mgbenka, & Mba, 2016). Smallholder farmers acknowledge that their farming alone is not able to provide them with the income they need to

meet their daily needs, so some of them learn skills to complement with income from farming. Two factors are responsible for this; first, smallholder farmers have a limited plot of land to cultivate on. In the second place, they use only labour from family members. In many communities, smallholder farmers acquire art skills with the expectation that it will assist them to augment their income and improve their socio-economic ratings.

Although skill acquisition has been regarded in literature (Udo 2015; Fatok 2019; Inyang & Agwadu 2017) as an important requirement for economic empowerment, if such skills are not marketed, they will not serve the interest of those who possess them. This situation is likened to a production process where it is said that production is not complete until it gets to the final consumer. Boxall *et al.* (2019) affirm that skill utilization is one of the surest ways that enable those with such skills to benefit from the skills they possess. Studies from different countries such as Brazil (Tiago *et al*., 2010), China (Li, 2018), Malaysia, (Ekpe, *et al*., 2015) and Mexico (Atkin 2016) have all pointed to the fact that skill acquisition is essential for economic empowerment of people.

Within the context of this study, it can be said that when a person possesses a skill set, it is expected that such skills will assist him or her in the area of income growth. This is because skills are essential assets and those who possess them expect to benefit from such. Atkin (2016) affirms that there is a significant relationship between skill possession and economic growth. Li (2018) holds a similar view regarding the usefulness of skill acquisition in promoting economic growth.

Researchers (Gever *et al.,* 2021; Okpara *et al.*, 2021) pay greater attention to investigating issues related to technical skill acquisition as though skill acquisition by itself will automatically result in an improvement in income and the earning capacities of those with such skills. According

to Tokarčíková *et al.* (2020), entrepreneurial competence is essential for people to survive as well as market their potential with a view to improving their economic abilities. Omolara (2018) in making a case for the centrality of entrepreneurial competence says that one of the reasons businesses in Nigeria find it difficult to survive is because business owners do not have the required skills to operate effectively in a competitive environment. Bosire and Nzaramba (2013) in making a case for the centrality of entrepreneurial competence aver that such skills will make people self-dependent and economically viable. Global Entrepreneurial Monitor (2012) cited in Omolara (2018) says that when it comes to business spirit, Nigeria ranks high, but instances of failed business are equally high in Nigeria. Omolara attributes such instances to low entrepreneurial competence. Small group communication is one of the approaches that can be used to improve the entrepreneurial competence and economic self-efficacy of smallholder farmers with art skills. This is because small group communication allows people interact and exchange ideas in a close range. Small group communication enables people to set goals, pursue such goals, outline how to achieve the goals and evaluate their performance. Naughton (2006) affirms that small group communication is an effective strategy that promotes interaction and knowledge internalization. From the submission of Naughton, it can be argued that small group communication can serve as tool for guiding smallholder farmers to convert their skills to money. Therefore, the objective of this study was to develop and evaluate the impact of small group communication programme on the economic self-efficacy of smallholder farmers with art skills in Nigeria.

Study Objective and Significance

The basic objective of this study was to develop and evaluate the impact of small group communication programme on the economic self-efficacy of smallholder farmers with art skills in Nigeria. A study of this nature is important to literature because previous studies have paid more

attention to the importance of skill acquisition with less attention to how acquired skills can be marketed. Therefore, the current study has addressed this gap. Furthermore, this study has practical relevance because policymakers will find it useful as a guide for promoting the marketing of skills. This is important because if possessed skills are not monetized, they may not be of economic value to those who possess them.

Effect of Small Group Communication

Communication is central to business promotion. Without communication, it will be difficult for any meaningful business engagement to take place. Communication is at the heart of all marketing promotions. These are advertising, sales promotion, personal selling, publicity, and direct marketing respectively. It will be difficult, if not impossible for any of the marketing promotions to take place without the use of communication. According to Scott and Walker (2008), promotional activities have proven to be essential in ensuring the success of department stores in fending off competition from the expanding chain stores by drawing in customers to their large, central premises. The point to make here, therefore, is that communication is at the centre of business activities. Communication can take place under different circumstances involving different people. For example, when the communication involves two persons on a face-to-face basis, it is called interpersonal communication (Ezeah *et al*., 2020). When it involves the use of mass media to a scattered, large, anonymous and heterogeneous audience, it is called mass communication (Gever 2016). Communication can also involve three or more persons who engage in a communication exercise to achieve a particular objective. Such communication is called small group communication.

One of the pioneers of small group communication is Bormann and Bormann (1980). According to Bormann and Bormann, small group communication describes the exchange of meaning involving two or three persons who have a particular objective to attain within a certain time frame. From the views of Bormann and Bormann, small group communication has two important elements. The first is that small group communication has a goal that participants wish to achieve. The second element is that such goals will be achieved within a certain time frame. The implication here is that small group communication has the potential to assist people to achieve goals. David and Chris (2009) say small group communication occurs on a face-to-face or any other channels of communication in accordance with the channels of communication agreed on among participants. According to Fujishin (2007), small group communication starts by first determining those who are involved in the communication engagement. When a membership is determined, it gives way for interaction among group members, which typically occurs in two dimensions. The first dimension is the task dimension which focuses on the events that will take place in the communication. Within the context of the task dimension, group members engage in some activities with the objective of ensuring that the goals of the group are achieved. The second dimension is the social dimension which describes the social relations that occur among group members in the course of the communication. According to Burtis and Turman (2006:105), small groups mostly develop and utilize patterns aimed at providing direction for their communication engagement which Burtis and Turman (2006) christened communication networks. The fundamental thing about small group communication is that it is driven by goals. Within the context of this study, the goal of using small group communication will be to improve entrepreneurial competence and economic self-efficacy.

Small group communication has the potential to serve as an effective tool for behaviour change. Gever *et al*. (2021) conducted a study to assess the impact of small group communication in influencing behaviour intention regarding art skills. The researchers utilized a quasi-experiment design to achieve their objectives. They examined 470 respondents who were victims of conflict. The art skill that was used in the study was painting while the researchers applied a *t*-test to analyse the results of the study. They found that small group communication was an effective communication tool for promoting skill acquisition among victims of conflict. Communication at the family level as well as family support to smallholder farmers with art skills are important considerations when discussing use of art skills for economic benefits. Akhlaq *et al*. (2013) in a study reported that family communication plays a crucial role in creating an atmosphere of positive change and growth among family members. On the other hand, family support is an essential condiment that encourages progress among family members (Schrodt 2009).

Madonsela (2010) carried out a study in which they tested the impact of small group communication in assisting formal organizations to achieve their goals. The researchers utilized survey research design and reported that small group communication is capable of aiding organizations to achieve their objectives. The result of the study suggests that small group communication is an effective behaviour change tool. Lowry *et al*., (2006) conducted a study wherein they ascertained the effect of different group sizes as well as social presence on small-group communication. The design that was used for the study was a quasi-experiment with a questionnaire as the instrument for data collection. The result of the study showed that small group communication is an effective behaviour change tool. Another study worth mentioning here is that of Lamport and Rynsburger (2008) who reported that small group communication is an efficient tool for evangelism. The critical point to note from the above-reviewed studies is that small group

communication has the potential to improve the entrepreneurial competence and economic self-efficacy of smallholder farmers with art skills.

Art skills among farmers, entrepreneurial competence, and economic self-efficacy

In most traditional African societies, smallholder farmers also possess art skills to enable them to meet their needs for some items. Art skills describe the ability to create work of art that will be of benefit to mankind. Examples of some art skills include drawing, weaving, musical composition, sculpting and painting. Smallholder farmers who possess art skills combine it with farming with the expectation that this will assist them to leave a better life. However, their low knowledge of entrepreneurial principles makes it difficult for them to maximize the potential inherent in the skills they possess. Troncoso and Vergara (2013) reveal that from time immemorial, farmers engage in art making as a deliberate strategy to diversify their income and improve their standard of living. Kei and Line (2022) affirm that many rural farmers acknowledge the importance of art in their lives.

Entrepreneurship has been regarded as critical to the economic development of any nation. Melugbo *et al*. (2020) affirm that entrepreneurship is the life-wire of economic development in any nation. Conceptually, entrepreneurship entails starting and managing a business entity. According to Barot (2015), entrepreneurship is important to everybody and anyone who begins a new business entity and is venturing into the world of entrepreneurship. According to Chang and Wyszomirski (2015), art entrepreneurship is gradually becoming common and it entails converting knowledge of artwork into business ventures.

Suffice it to say that entrepreneurship entails engagement in business activities, then it follows logically that entrepreneurial competence is important for successful entrepreneurial

activities. Such skills are needed so that entrepreneurs will be successful at what they do. Evidence in the literature (Khedhaouria, *et al*. 2015; Salman *et al*. 2016) points to the fact that entrepreneurship competence is an essential requirement for successful entrepreneurial activities. Khedhaouria, *et al*. (2015) say that entrepreneurial competence, not certificate, determines the success of businesses.

When entrepreneurs develop competence, it is expected that such skills will help to positively impact economic self-efficacy. That is to say that it is expected that the possession of entrepreneurial competence will translate to success in business and eventually income. Economic self-efficacy can be defined as the ability of a person to meet their financial needs independent of other people. Hoge *et al*. (2017) note that economic self-efficacy is important because it allows people to meet their daily needs while also maintaining a sense of self-worth. Elise and Ellen (2022) opine that economic self-efficacy plays a significant role in influencing the type of relationship that exists among people. The central point to note here is that the economic self-efficacy of smallholder farmers is crucial in determining their standard of living, hence the need to suggest ways of improving their economic self-efficacy.

Theoretical framework

The researchers applied the Human Capital Theory (HCT) to conduct the current study. Gary Stanley Backer propounded the human capital theory in 1964 to explain the importance of skill acquisition in empowering people (Goldin 2014). The basic assumption of the human capital theory is that skill sets are just as important as money such that when people acquire saleable skills, it helps them to improve their economic fortunes. The theory assumes that one of the ways to

promote economic development is by equipping people with skills that will make them self-dependent and sustaining.

Generally, researcher (Inyang & Agwadu, 2017; Okpara *et al*., 2021) agree that skill acquisition is an important strategy for empowering people. For example, Okpara *et al*. in a study reported that when people acquire skills, it is likely to improve their economic status than those without saleable skills. Obasi *et al.* (2021) applied the human capital theory to ascertain the impact of theatre for development in improving the acquisition of skills in painting and weaving among a sample of 470 victims of conflict. The result of the study showed that the theory was an appropriate framework for assessing issues related to skill acquisition. Okpara *et al*. (2021) equally applied the story to examine the acquisition of skills among jobless youth in Nigeria and reported that the framework provides a sufficient basis for understanding the importance of skill acquisition and how people can acquire skill sets. The argument that the human capital theory advances suits very well with the current study is because when people are equipped with entrepreneurial competence, it will have a corresponding positive effect on their economic self-efficacy. When people acquire skills without using them, the essence of human capital development is defeated. The theory, thus, assumes that people who are equipped with skills will use such skills to make life better for themselves. Smallholder farmers with art skills need to make use of such skills so that they can actually be empowered. The development of human capital requires the use of appropriate tools. Within the context of this study, small group communication is regarded as a useful tool for developing the human capital of smallholder farmers. According to Gever *et al*. (2021), small group communication is an effective tool for assisting individuals to develop skills aimed at making them economically useful to themselves and their immediate environment. Arias *et al*. (2016) conducted a study wherein they compared small group communication with lecture format

and reported that the formal was a far more effective strategy for promoting skill acquisition than the lecture format.

Family characteristics are essential when assessing acquisition skills and how such skills will help empower people. This is so because the family is the immediate surrounding of everyone, and events that take place at the family level could impact essential aspects of individuals. Nocentini *et al*. (2019) say that family functioning and the relationship that exists among family members play a significant role in influencing people's behaviour. Akhlaq *et al.* (2013) say that usually, a family is a dynamic, self-correcting, goal-directed and interconnected system that affects its members significantly. There are families where members freely relate with each other and openly communicate among themselves. In addition, there are families where open communication is lacking among other members. Communication is the verbal and non-verbal exchange of ideas among family members. Peterson and Green (2009) note that communication enables family members to resolve disagreements and remain united. Another vital family characteristic is family support. It can be described as the material and non-material assistance that family members give to each other. Pinkerton and Dolan (2007) in a study reported that family support is an important determinant in building resilience during challenging times. Based on this theory, the researchers hypothesized:

H1: Smallholder farmers who take part in a small group communication intervention will score higher means regarding their entrepreneurial competence than those who do not.

H2: Smallholder farmers who take part in a small group communication intervention will score higher means regarding their economic self-efficacy than those who do not.

H3: Family characteristics such as open family communication and family support contribute to effective use of art skills for economic benefits among smallholder farmers.

Methodology

Study 1:

In the first study, the researchers sought to develop a small group communication programme that could be used to improve the entrepreneurial competence and economic self-efficacy of smallholder farmers with art skills. Therefore, content analysis was the design of the study. The participants (n=33) were experts in agricultural economics (n=8), mass communication (n=7), counseling (n=7), social work (n=5), and entrepreneurship (n=6). The researchers recruited the participants using the respondents-driven chain referral sampling technique. This was done by first recruiting earlier participants who were requested to recommend potential participants. The instrument of data collection was a face-to-face interview that lasted between 30 minutes to one hour. Each interview began with a broad question- how can small group communication be used to improve entrepreneurial competence and economic self-efficacy of smallholder farmers with art skills? Follow-up questions were asked to guide the interview session. Based on the interview session, Figure 1 reveals a developed programme for improving entrepreneurial competence and economic self-efficacy of smallholder farmers with art skills.

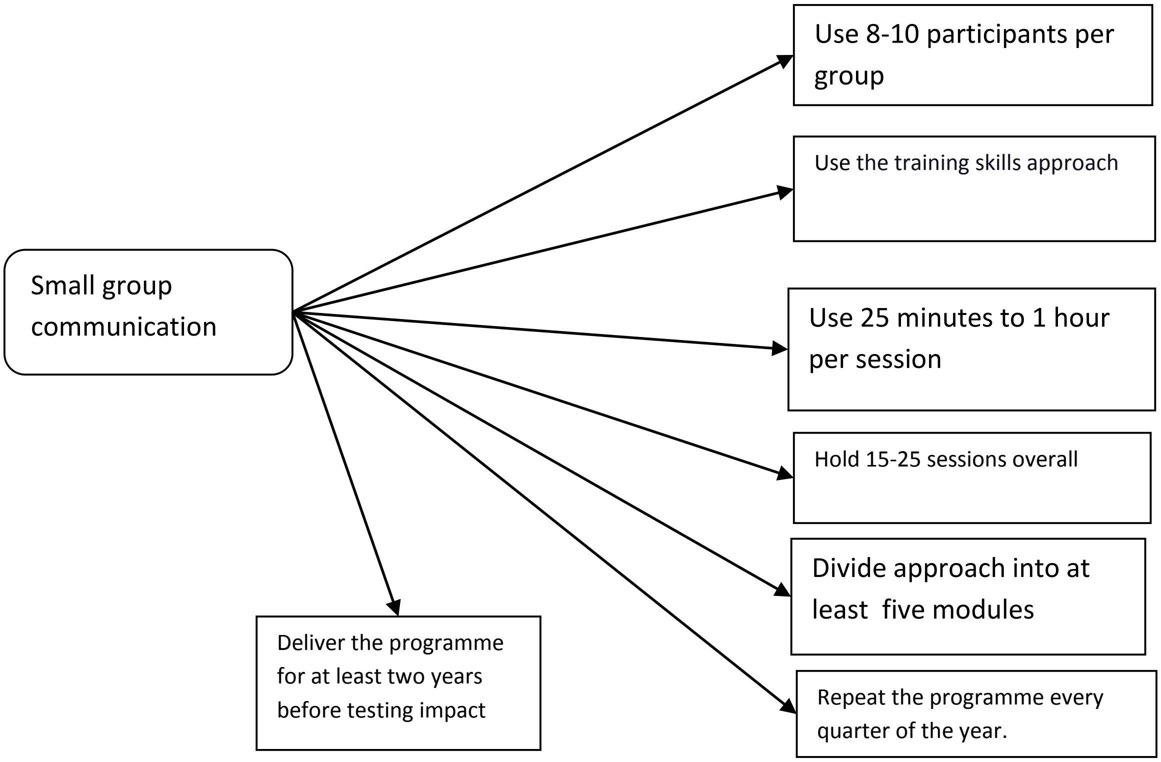

Figure 1: A programme for using small group communication to improve entrepreneurial competence and economic self-efficacy of smallholder farmers with art skills

In Figure 1 above, the researchers present a small group communication programme that could be used to improve the entrepreneurial competence and economic self-efficacy of smallholder farmers. It is important to clarify here that the training skills take the education approach where the focus is to educate the participants on an issue.

Study 2

In study 2, the researchers attempted to test the effectiveness of the developed programme. The design that was used in this study was a quasi-experiment. A quasi-experimental design is mostly required when a non-laboratory experiment is needed to test the effectiveness of an intervention (Obasi *et al*., 2021; Okpara *et al*., 2021). The participants in this study were 540 smallholder

farmers with art skills. Before deciding on the sample size, the researcher carried out a priori power analysis using the G*power programme. The output from this analysis showed that the researchers needed a sample size of 540 to determine the existence of a relationship at 0.05 level of significance. The study was carried out in Ede-Oballa, in Nsukka Local government area of Enugu State, Nigeria. The area has two autonomous communities namely: Ede-Ukwu and Ede-Enu. Combined, both communities have fifty-two villages, twenty-two from Ede-Enu and thirty from Ede-Ukwu. The study announcements were made at the village halls and those interested were asked to fill forms and submit. The forms sought information on the art skills, farming activities, family communication and family support of those who indicated interest. The information submitted was evaluated by a team of 10 research administrators. The team was made up of two experts each in measurement and evaluation, fine and applied arts, communication, agricultural economics and business education. The mandate of the research administrators was to ensure that the right participants were included in the sample. Initially, 1500 indicated interest to participate, but during screening, only 1,080 participants were eligible. The researchers needed only 540 participants so systematic sampling technique was used. This was done using the following systematic approach:

1. The smallholder farmers were numbered from 1-1080.
2. The sample size, n was decided to be 540.
3. The interval, k=2. That is 2 × 540=1,080 and $\frac{1080}{540} = 2$

Every second participant was selected and this continued until all the 540 participants were sampled. The participants were then randomly assigned to control and treatment groups made up of 270 participants each.

The researchers used entrepreneurial competence scale and economic self-efficacy scale to collect data for the study.

Entrepreneurial competence scale: This scale was developed by Silveyra *et al*. (2020) to assess economic competence. It has nine items spread across three dimensions namely competence in identifying opportunities (3 items; α= 0.88), evaluation of identified opportunities (3 items; α= 0.89), and exploitation of opportunities (3 items; α= 84). The overall reliability was .87. Also, a pilot study with 30 participants showed an overall Cronbach's alpha of .77, meaning that it was okay for the Nigerian sample.

Economic Self-Efficacy Scale: Hoge *et al.* (2017) developed this scale with 10 items to assess the financial independence of individuals. Hoge *et al*. reported a Cronbach's reliability coefficient of .88. The researchers also conducted a pilot study in Nigeria with 30 participants and this revealed a Cronbach's reliability coefficient of .86 which was regarded as acceptable.

Procedure for the experiment

To conduct this experiment, participants in the treatment group took part in a small group communication intervention. There were 27 small groups made up of 10 members each. Each group had a moderator who was an expert in entrepreneurship. The discussion focused on developing the entrepreneurial competence of respondents. The respondents organized the discussion in six modules that were organized based on entrepreneurial competence. In module one, the attention was on the importance of entrepreneurship. In the second module, the discussion focused on farming and art skills as entrepreneurial ventures. In the third module, attention was paid to the appropriate entrepreneurial competence. The fourth module was on how to become successful entrepreneurs while the fifth module was on challenges of entrepreneurship. Module

six focused on how to overcome challenges associated with entrepreneurship. Each of the modules was delivered three times, making it a total number of 18 sessions each year and 36 sessions for the two years. The researchers collected data for the study three times. The first was before the intervention, the second was after the intervention, while the last time was two years follow-up assessment.

Data Analysis

To conduct the analysis in this study, the researchers utilized Statistical Package for Social Sciences version 22. Furthermore, the researchers deployed simple percentages, mean and standard deviation as well as a two-way analysis of variance (ANOVA) with repeated measures to explore the impact of the programme at pre-stage and post treatment.

Ethical Approval

Ethical approval was sought and received from XXXX. The type of approval was exempt because participation in the study did not expose the respondents to danger.

Results

The return rate for this study was 94% for the control group and 92% for the treatment group. All the respondents were smallholder farmers with art skills. This means that the respondents had art skills that they could combine it with farming to make their lives better. The mean age of the respondents was 33 years. The result of Table 1 revealed the impact of the programme on the entrepreneurial competence and economic self-efficacy of smallholder farmers with art skills.

Table 1: Pre-intervention, post-intervention and follow-up ANOVA results on entrepreneurial competence and economic self-efficacy of smallholder farmers with art skills

	Entrepreneurial competence					*Economic self-efficacy*				
	Mean	***SD***	***P***	***F***	***Dec.***	**Mean**	**SD**	***P***	***F***	**Dec**
**Pre-intervention**										
Control group	16.5	3.8				10.6	1.5			
Treatment group	16.4	3.9				10.6	2.3			
			.134	2.258	Not sig			.914	. .012	Not sig
**Post-intervention**										
Control group	16.4	3.6				10.8	1.5			
Treatment group	37.7	3.5				37.3	3.7			
			.001	436.117	sig			0.001	11656.8	Sig
**Follow-Up assessment**										
Control group	16.5	3.8				11.1	2.5			
Treatment group	38.3	3.7				30.7	4.0			
Total average			.001	4272.751	sig			0.001	4471.76	Sig

Table 1 was computed to ascertain the entrepreneurial competence and economic self-efficacy of smallholder farmers who took part in the study. The result of the study showed low entrepreneurial competence and economic self-efficacy among the participants with no significant difference

detected between the treatment and control group at pre-treatment. After the programme, participants in the treatment group reported a significant improvement in their entrepreneurial competence and economic self-efficacy, but those in the control group did not report a significant improvement even after a follow-up assessment after two years. The result achieved statistical significance even after controlling for type of art skills ($p=0.004$). Based on this result, hypotheses 1 and 2 were supported. The graphical illustration of the result is further presented in Figures 2 and 3 below:

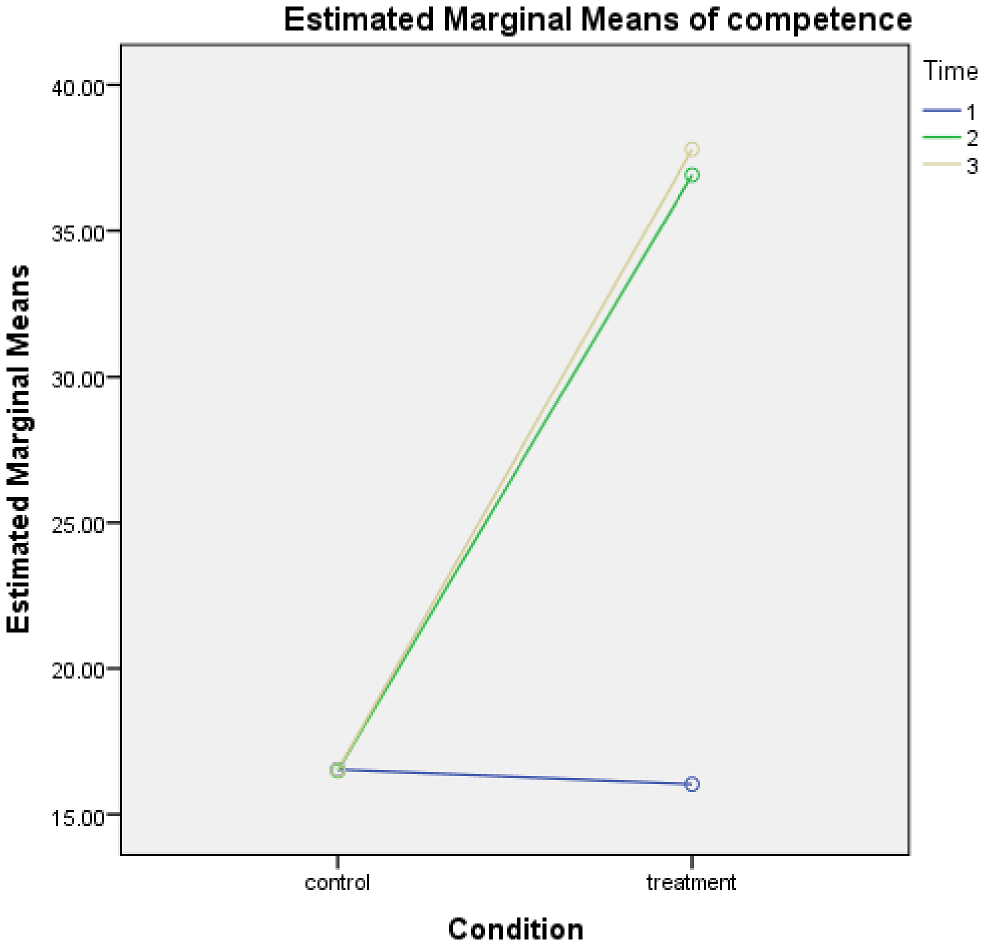

Figure 2: Entrepreneurial competence of the participants at before, after and follow-up assessment

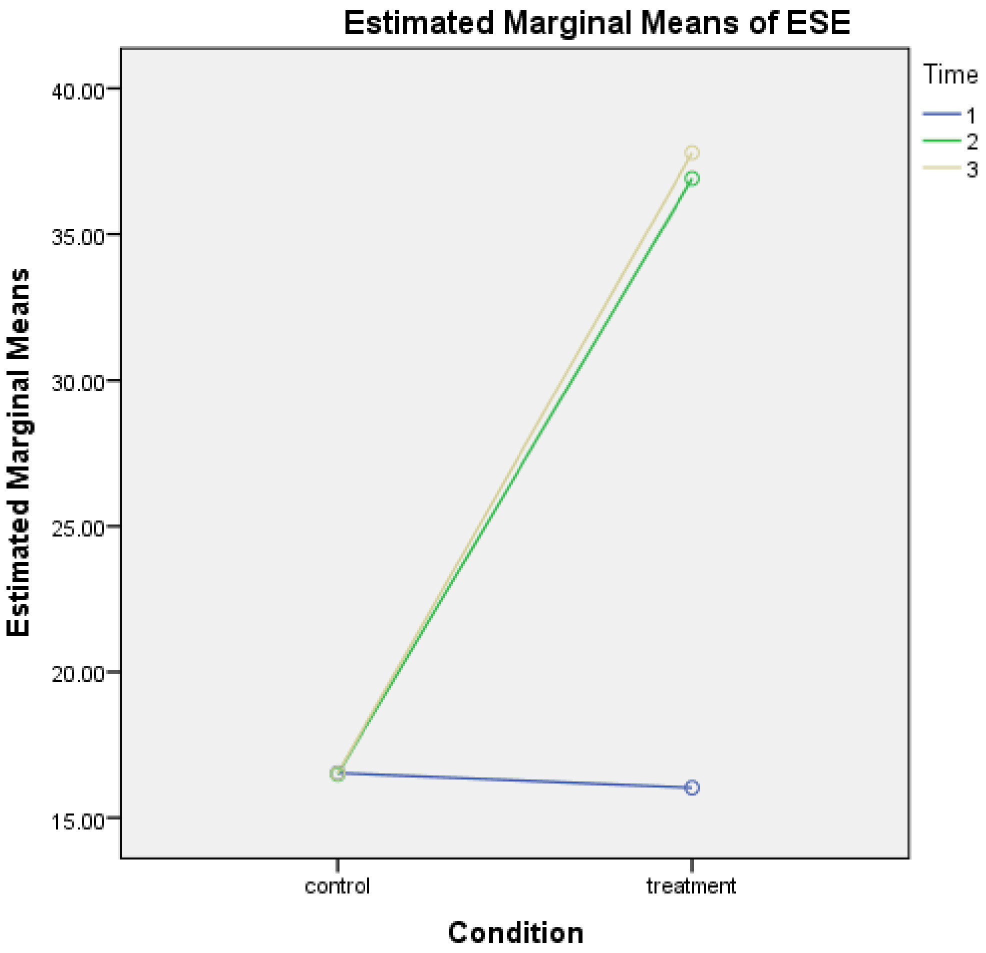

Figure 3: Economic self-efficacy of the participants before, after and follow-up assessment

Table 2: Regression analysis of the predictive power of open family communication and family support on use of art skills for economic benefits

	Constant	*β value*	*R square*	*F. value*	*P. value*
Open family communication	2.201	.381	.334	52.521	.001
Family support		.252			.001

The researchers computed Table 2 to determine the contributing role of family characteristics such as open family communication and family support on the effective use of art skills for economic benefits. The results showed that the model contributes 33.4% in the contributing role of family characteristics on harnessing the economic benefits of art skills among smallholder farmers, R^2=.334, p=0.001, *F*(3,211) 52.521. Comparatively, open family communication (β=.381) contributed more than family support. (β=.252).

Discussion of Findings

In this study, the researchers set out to develop and measure the effectiveness of a small group communication programme in improving the entrepreneurial competence and economic self-efficacy of smallholder farmers who possess art skills. Two independent but complementary studies were conducted. The first study was an exploratory effort to develop a small group communication programme for improving the entrepreneurial competence of smallholder farmers with art skills. After the programme was developed, its effectiveness was tested on a sample of 540 smallholder farmers with art skills in the area of weaving, sculpture and music. The result of the study showed that before the treatment was administered to the respondents, both the treatment and the control groups scored low regarding entrepreneurial competence and economic self-efficacy. What this means is that even though smallholder farmers acquire art skills, they lack

entrepreneurial competence and are unable to improve their economic self-efficacy. This highlights the need to assist them so that they will be able to increase their earnings. It is not sufficient to possess skills; such skills must be able to put food on the table of those who possess them. Okpara *et al*. (2021) in a study reported that many people with art skills do not have jobs. Accordingly, they argued that entrepreneurship, especially in arts is one of the ways that people with art skills can improve their income. Gever *et al.* (2021) in their study also reported that art skills, when properly applied, can be useful in economic empowerment.

Furthermore, the result of this study showed that after the intervention, there was a significant mean difference between the control and the treatment groups. In particular, it was found that respondents in the treatment groups scored higher regarding their entrepreneurial competence and economic self-efficacy. What this means is that small group communication played a useful role in assisting participants to improve their skills as well as income. This result has extended studies (Lowry *et al*., 2006; Lamport & Rynsburger, 2008; Madonsela, 2010) related to the effectiveness of small group communication by adding how it can play a critical role in entrepreneurship and income. This is because most of the previous studies on the effectiveness of small group communication pay less attention to issues related to income. Additionally, the result of the current study has extended previous studies ((Inyang & Agwadu, 2017; Okpara *et al.*, 2021) related to skill acquisition by pointing out the need to convert acquired skills into income. It is one thing to acquire skills and another to ensure that such skills are useful for those who acquire them. Another addition in this study that is worth pointing out is the contributing role of family characteristics such as open family communication and family support in predicting the use of art skills for economic benefits. This part of the result has extended the studies of Akhlaq *et al*. (2013)

and Schrodt (2009) who highlighted the centrality of the family in promoting positive development without looking at art skills of smallholder farmers.

Implications of the Results

The result of this study has three broad implications namely: scholarship, theory and practice. Scholarly, this study has extended the argument regarding the importance of small group guidance communication as an intervention tool, the need for entrepreneurial competence as well as how this is linked to economic self-efficacy. This new dimension which links small group guidance communication with income and entrepreneurial competence will shape future debates in the literature on communication effectiveness, business, and art skills.

The result of this study also has implications for the connection between art skills and entrepreneurial ability. Art skills are good but they may not be able to serve those who possess them if they do not have the corresponding entrepreneurial competence to market those skills. Skills are assets and can only be useful to those who possess them if they are able to convert them into money. Therefore, this study has shown that there is a need to train those with technical skills in the area of entrepreneurship so that they will benefit from the skills that they have.

Furthermore, the result of this study has implications for the human capital theory by showing that human capital development should not be limited to skills acquisition but also extended to the utilization of such skills to make money. This is important because skills on their own do not translate to anything if they are not maximized to the benefit of those who possess them. This aspect of the current study has added a new perspective to the human capital theory by showing that programmes are needed to move from skill acquisition to skill utilization.

The result of the current study has practical implications by highlighting the need for change agents in both government and non-profit organizations to utilize small group communication as behaviour change tool. This is because small group communication allows for close interaction among people. It offers people the opportunity to ask questions and receive answers. This is not possible in mass media of communication.

Overall, this study has filled a gap in the literature by going beyond examining the importance of skill acquisition by providing empirical evidence that could be useful in developing learning packages for assisting smallholder farmers to improve their economic self-efficacy.

Conclusion/ suggestions for further studies

Based on the result of this study, the researchers conclude that small group communication can serve as a useful tool for assisting smallholder farmers to acquire entrepreneurial competence and improve their income. The study equally concludes that family characteristics such as family communication and support are essential determinants in assisting smallholder farmers with art skills to make economic gains from their skills. The basic contribution of this study is that it has provided empirical evidence that not only explains the impact of small group communication in improving business performance but has highlighted the need to encourage smallholder farmers with art skills to convert their skills into money. Despite this, the current study has some limitations. First, the researchers examined only smallholder farmers, it will be beneficial to examine other categories like university graduates with certification in vocational and technical skills. The researchers did not also examine other skill sets apart from arts. Further studies should look at these limitations. It is also suggested that further studies should apply social exchange theory to examine the impact of small group guidance communication on behaviour change.

Conflict of Interest

We declare no conflict of interest

Funding

We did not receive funding for this study.

References

Ale, V. (2020). A library-based model for explaining information exchange on Coronavirus disease in Nigeria. *Ianna Journal of Interdisciplinary Studies*, 2, (1)1-11.

Akhlaq, A. Malik, N. & Khan, N (2013). Family communication and family system as the predictors of family satisfaction in adolescents. *Science Journal of Psychology, 1, 1-6*

Arias, A., Scott, R., Peters, O. A., McClain, E., & Gluskin, A. H. (2016). Educational outcomes of small-group discussion versus traditional lecture format in dental students' learning and skills acquisition. *Journal of Dental Education*, 80(4), 459–465. doi:10.1002/j.0022-0337.2016.80.4.tb06104.x

Atkin, D. (2016). "Endogenous skill acquisition and export manufacturing in Mexico." *American Economic Review*, 106 (8), 2046-85.DOI: 10.1257/aer.20120901

Barot, H. (2015). Entrepreneurship - A key to success. *The International Journal of Business and Management,*3(1), 163-165.

Bormann, E. G. & Bormann, N. C. (1980). *Effective small group communication*. (3rd ed). Burgess.

Bosire, K., & Nzaramba. (2013). Entrepreneurship skill development and growth of small and medium enterprises in Rwanda. *Journal of Information Technology and Business Management*. 17(1), 12-25.

Burtis, J. O. & Turman, P. D. (2006). *Group communications pitfalls: Overcoming barriers to an effective group*. (3rd ed). SAGE

Boxall, P., Huo, M.-L., & Winterton, J. (2019). How do workers benefit from skill utilisation and how can these benefits be enhanced? *Journal of Industrial Relations*, 61 (5)1-12 002218561881916. doi:10.1177/0022185618819169

Chang, W.J., Wyszomirski, M. (2015). What is arts entrepreneurship? tracking the development of its definition in scholarly journals. *Journal of Entrepreneurship in the Arts*, 4, (2),11-31.

David, W. W. & Chris, R. K. (2009). An introduction to the study of small group communication. [Web:] www.flipkart.com/communication-small-groups-john-cragan-book-0495793892

Ekpe, I., Che, R., Razali, I., Mohammad, A. (2015). Entrepreneurial Skill Acquisition and Youth's Self-Employment in Malaysia: How Far?. *Mediterranean Journal of Social Sciences, 6 (4),150-154.* doi:10.5901/mjss.2015.v6n4p150

Elise, F. F., & Ellen, K. N. (2022). Financial self-efficacy, financial literacy, and gender: A review. Journal of Consumer Affairs https://doi.org/10.1111/joca.12436

Ezeah, G. Okumba, O. Ohia, C. & Gever, V. C. (2020). Measuring the effect of interpersonal communication on awareness and knowledge of COVID-19 among rural communities in Eastern Nigeria *Health Education Research.* 35 (5), 481–489. doi:10.1093/her/cyaa033

Fatoki, O. (2019). The skills acquisition programme and youth empowerment in Ondo State of Nigeria: An empirical study. *Global Journal of HUMAN-SOCIAL SCIENCE: A Arts & Humanities – Psychology*, 19 (6),6-15

Fujishin, R. (2007). *Creating an effective group: The art of small group communication*, (2nd ed). Inter-Varsity Press.

Faul, F., Erdfelder, E., Buchner, A., & Lang, A. G. (2007). G*Power 3: A flexible statistical power analysis program for the social, behavioral, and biomedical sciences. *Behavior Research Method*, 39 , 175–191.

Gever, V. C., Talabi, F., Anibueze, A., Okparsa, C., Ugwuoke, J. (2021). Effect of small group communication counseling intervention strategy on knowledge and intention towards painting among Internally Displaced Persons of the farmers/herdsmen conflict in Benue State, Nigeria. *Journal of Refugee Studies*. https://doi.org/10.1093/jrs/feaa132

Gever, V. C. (2016). Questioning the safe school initiative and making a case for a safe Nigeria model: The media as the nucleus. *Sokoto Journal of Social Sciences* 5 (6)1-16.

Goldin, C. (2014). Human Capital. In Claude Diebolt & Michael Haupert, (Eds) (2014): Handbook of Cliometrics. Harvard: Department of Economics, Harvard University and National Bureau of Economic Research -1- Springer-Verlag.

Handcock, M & Gile, K. (2011). Comment: On the concept of snowball sampling. *Sociological Methodology*, 41(1), 367-371

Hoge, G. L., Stylianou, A. M., Hetling, A., & Postmus, J. L. (2017). Developing and validating the scale of economic self-efficacy. *Journal of Interpersonal Violence, (), 088626051770676*–. doi:10.1177/0886260517706761

Inyang, M. N. & Agwadu, D. K. (2017). Empowering the rural poor through vocational skills acquisition: Nasarawa state in focus. International *Journal of Development and Sustainability*, 6(3) 115-129.

Kari, K. (2020). Predictors of the utilization of digital library features among women patrons: The moderating role of marital status. *Ianna Journal of Interdisciplinary Studies*, 2, (1),86-94.

Kei, L. & Line, M. (2022). Experiencing art from a field of rice: How farmers relate to rural revitalisation and art at Japan's Echigo-Tsumari Art Festival. Sociologia Ruralis, DOI: 10.1111/soru.12379

Khedhaouria, A., Gurău, C., Torrès, O. (2015). Creativity, self-efficacy, and small-firm performance: The mediating role of entrepreneurial orientation. *Small Business. Economics*, 44, 485–504.

Lamport, M. & Rynsburger, M. (2008). All the rage: How small groups are really educating Christian adults part 2: Augmenting small group Ministry practice—developing small group leadership skills through insights from cognate theoretical disciplines. *Christian Education Journal*,5(3), 391- 413.

Li, B. (2018). Export expansion, skill acquisition and industry specialization: Evidence from China. *Journal of International Economics, 114,* 346-36, *S0022199618302216*–. doi:10.1016/j.jinteco.2018.07.009

Lowry, P. B., Roberts, T. L., Romano, N. C., Cheney, P. D., & Hightower, R. T. (2006). The impact of group size and social presence on small-group communication. *Small-Group Research, 37(6), 631–661.* doi:10.1177/1046496406294322

Madonsela, N. A. (2010). The role of small group communications in a corporate working environment. https://pdfs.semanticscholar.org/6484/dc953ea1233bd96629fb082f1292f27c4f19.pdf

Melugbo, D. U., Ogbuakanne, M. U., & Jemisenia, J. O. (2021). Entrepreneurial potential self-assessment in times of COVID-19: Assessing readiness, engagement, motivations and limitations among young adults in Nigeria. *Ianna Journal of Interdisciplinary Studies* , *2*(1), 12-28.

Mgbenka, R. N & Mba, E. (2016). a review of smallholder farming in Nigeria: need for transformation. *International Journal of Agricultural Extension and Rural Development Studies,* 3(2).43-54.

Naughton, D. (2006). Cooperative strategy training and oral interaction: Enhancing small group communication in the language classroom. *The Modern Language Journal*, 90(2), 169–184. doi:10.1111/j.1540-4781.2006.00391.x

Nocentini, A., Fiorentini, G., Di Paola, L., & Menesini, E. (2019). Parents, family characteristics and bullying behavior: A systematic review. *Aggression and Violent Behavior*. 45, 41-50 doi:10.1016/j.avb.2018.07.010

Obasi, T., Okpara, C., Okpara, F., Itiav, V. & Gever, V. C. (2021). Effect of theatre for development as a communication intervention strategy on behavioural intentions towards painting, weaving and fashion and design among victims of conflict in Nigeria, *African Security Review*, DOI: 10.1080/10246029.2020.1857805

Okpara, C.V., Anibueze A., Ugwu, A. Ugwuanyi J.C., & Gever, V. C. (2021). Modelling the effectiveness of a social media-based counseling intervention on interest, entrepreneurial skills, and behavioural intention towards painting among youth without paid jobs in Nigeria. *Vision-Journal of Business Perspective*

Omolara, A. (2018). A comparative analysis of Nigerian entrepreneurs and minority entrepreneurs in the UK. Retrieved from https://mpra.ub.uni-muenchen.de/86751/1/MPRA_paper_86751.pdf

Peterson, R. & Green, S. (2009) Families first: Keys to successful family functioning communication. Virginia Tech: Virginia State University

Pinkerton, J., & Dolan, P. (2007). Family support, social capital, resilience and adolescent coping. *Child & Family Social Work*, 12(3), 219–228. doi:10.1111/j.1365-2206.2007.00497.x

Sabo, B.B, Isah, S.D., Chamo, A.M. & Rabiu, M.A. (2017). Role of Smallholder Farmers in Nigeria's Food Security. *Scholarly Journal of Agricultural Science*, 7(1), 1-5.

Salman, M. S., Mohd, N., Mohd, S., & Arfan, S. (2016), Determinants of entrepreneurial skills set in Pakistan: A Pilot Study, *International Journal of Academic Research in Accounting, Finance and Management Sciences*, 6, (2) 76–86.

Scott, P. & Walker, J. (2008) Advertising, promotion, and the competitive advantage of interwar UK department stores. Retrieved from https://www.researchgate.net/profile/Peter_Scott16/publication/227372808_Advertising_Promotion_and_the_Competitive_Advantage_of_Interwar_British_Department_Stores/links/00b7d5363618e48fbb000000.pdf?origin=publication_detail

Schrodt, P. (2009). Family strength and satisfaction as functions of family communication environments. *Communication Quarterly*, 57, 171-186.

Silveyra, G., Herrero, Ã., & Parez, A. (2020). Model of teachable entrepreneurship competencies (M-TEC): Scale development. *The International Journal of Management Education, (), 100392–.* doi:10.1016/j.ijme.2020.100392

Tiago, C., Guimaraes, J. & Sampaio, B. (2010). Barriers to skill acquisition in Brazil: Public and private school students' performance in a public university entrance exam. *,Quarterly Review of Economics and Finance, 50(4), 395407.* doi:10.1016/j.qref.2010.08.001

Tokarčíková, E., Malichová, E., Kucharčíková, A. & Durišová, M. (2020). Importance of Technical and Business Skills for Future IT Professionals. *Amfiteatru Economic*, 22(54),567-578.

Troncoso, A. & Vergara, F. (2013). History, landscape, and social life: Rock art among hunter-gatherers and farmers in Chile's Semi-Arid North. *Time and Mind, 6(1), 105–112.* doi:10.2752/175169713X13518042629379

Udo, P. (2015). Techniques for improving practical skill acquisition in vocational business education (VBE) for sustainable development in Nigeria. *European Journal of Research and Reflection in Educational Sciences*, 3(1),27-34.
