# Supplementary material for: Developing and evaluating the impact of a small group communication programme in improving the entrepreneurial competence and economic self-efficacy of smallholder farmers with art skills
Source: PLoS One. 2023 Nov 2;18(11):e0292640. doi: 10.1371/journal.pone.0292640 (PMC10621803; doi:10.1371/journal.pone.0292640)
Supplement: Attachment — Submitted filename: Response letter Plos one.docx [file pone.0292640.s002.docx]

Please include the following items when submitting your revised manuscript:

- A rebuttal letter that responds to each point raised by the academic editor and reviewer(s). You should upload this letter as a separate file labeled 'Response to Reviewers'.
- A marked-up copy of your manuscript that highlights changes made to the original version. You should upload this as a separate file labeled 'Revised Manuscript with Track Changes'.
- An unmarked version of your revised paper without tracked changes. You should upload this as a separate file labeled 'Manuscript'.

**Comments to the Author**

1. Is the manuscript technically sound, and do the data support the conclusions?

The manuscript must describe a technically sound piece of scientific research with data that supports the conclusions. Experiments must have been conducted rigorously, with appropriate controls, replication, and sample sizes. The conclusions must be drawn appropriately based on the data presented.

Reviewer #1: Yes

Reviewer #2: Yes

2. Has the statistical analysis been performed appropriately and rigorously?

Reviewer #1: Yes

Reviewer #2: Yes

3. Have the authors made all data underlying the findings in their manuscript fully available?

The [PLOS Data policy](http://www.google.com/url?q=http%3A%2F%2Fwww.plosone.org%2Fstatic%2Fpolicies.action%23sharing&sa=D&sntz=1&usg=AOvVaw0txrLazDj_2hFfsX8TT2cP) requires authors to make all data underlying the findings described in their manuscript fully available without restriction, with rare exception (please refer to the Data Availability Statement in the manuscript PDF file). The data should be provided as part of the manuscript or its supporting information, or deposited to a public repository. For example, in addition to summary statistics, the data points behind means, medians and variance measures should be available. If there are restrictions on publicly sharing data—e.g. participant privacy or use of data from a third party—those must be specified.

Reviewer #1: Yes

Reviewer #2: Yes

4. Is the manuscript presented in an intelligible fashion and written in standard English?

PLOS ONE does not copyedit accepted manuscripts, so the language in submitted articles must be clear, correct, and unambiguous. Any typographical or grammatical errors should be corrected at revision, so please note any specific errors here.

Reviewer #1: Yes

Reviewer #2: Yes

5. Review Comments to the Author

Please use the space provided to explain your answers to the questions above. You may also include additional comments for the author, including concerns about dual publication, research ethics, or publication ethics. (Please upload your review as an attachment if it exceeds 20,000 characters)

**Comment:** Reviewer #1: The paper suggests promising interventions to effectively improving entrepreneurial capacities and economic self efficacy of smallholder farmers. The writing style is clear and concise; however, some minor grammatical and spelling errors are present. The introduction provides a coherent overview of the objectives and scope of the paper, well-structured and provides a comprehensive examination of the small communication programme. The conclusion summarizes the results and recommendations of the paper succinctly.

**Response:** The manuscript has been proof-read thoroughly for grammar.

Reviewer #2: Dear Author,
**Comment:** First of all I would like to say that the study presents the results of original research. The Experimental study is important for such kinds of topics and the author was successfully targeted the right sample with proper methodology. However, the following comments are important to be considered to further

improve the current study:
**Comment**: 1- Specify what art skills does the author refers, the author has to specify it.

**Response**: This has been done and specified.
**Comment**: 2- The literature should better explain measurements of self-efficiency, entrepreneurial competencies and dimentions:
Read:
**Response:** We have done this and highlighted in red.

i. Developing and Validating the Scale of Economic Self-Efficacy

**Response**: This has already been cited in the document. See methodology and reference list.

Effects of Intellectual Capital on the Performance of Malaysian Food and Beverage Small and Medium-Sized Enterprises.

**Response**: This has been cited now in both the document and the reference list.

iii. Developing self-efficacy and career optimism through participation in communities of practice within Australian creative industries

**Response:** This has been cited in the reference and the document.
iv. Developing and testing a social media-based intervention for improving business skills and income levels of young smallholder farmers

**Response**: This has been cited in the document and reference list.
**Comment**: 3- The author needs to develop the conclusion and provides more details on his findings and contribution.

**Respond**: We have included a conclusion and the contribution of the study. This has been highlighted in red.
**Comment**: 4- It would be much better of the author provides contradicted literature and justify his findings in discussion section.

**Response**: This has been done. We have shown how the current study differs with the previous ones. This has been highlighted in red.
